# Comparative Study of Modified Silver Nitrate Staining for the Detection of *Helicobacter pylori*

**DOI:** 10.1097/PAI.0000000000001010

**Published:** 2022-02-18

**Authors:** Xiaoying Chu, Jian Xu, Li Niu

**Affiliations:** Department of Pathology, Zhongnan Hospital of Wuhan University, Wuhan, Hubei, People’s Republic of China

**Keywords:** *Helicobacter pylori*, modified silver nitrate staining, sensitivity, specificity

## Abstract

*Helicobacter pylori* (Hp) infection is associated with a variety of diseases, such as benign lesions, precancerous lesions, and malignant lesions, especially diseases in the digestive system. Most people with Hp infection have mild early symptoms that are not easily noticed. Therefore, the diagnosis and treatment of Hp infection is particularly important. At present, there are many methods to detection Hp infection, but there is a lack of effective detection method with high sensitivity and specificity. On the basis of the existing detection methods, the modified silver nitrate staining method in this study improved the sensitivity and specificity of Hp detection.

**Materials and Methods:**

We selected gastric antrum and gastric angle mucosal biopsy tissues from 60 inpatients that were archived in the Pathology Department of Zhongnan Hospital of Wuhan University from July to December 2020. An Hp immunohistochemical assay, histochemical assay kit (methylene blue), and modified silver nitrate staining were used to measure the Hp infection positivity rate.

**Results:**

Comparison of Hp sensitivity and specificity among the 3 methods showed that the modified silver nitrate staining method was the most excellent. The sensitivity of modified silver nitrate staining method was 98.3%, which is statistically significantly higher compared with the other 2 methods.

**Conclusion:**

The modified silver nitrate staining method for Hp detection is convenient and effective, and could be widely used for clinical Hp detection.

Helicobacter pylori (Hp) are gram-negative rod bacterium that mainly appears helical, unbranched, and flagellated.^1^ Hp is mainly present in the gastric mucosa.^2^ At present, the Hp infection rate is ∼50% of the world’s population, and many types of gastric tissue inflammatory diseases and neoplasm are associated with Hp infection.^2^,^3^ The gastric mucosa provides a sterile and highly acidic environment for Hp to survive. However, the most Hp-positive patients do not have any apparent clinical symptoms and replaced by the digestive ulcers and gastric neoplasm.^4^ With the rapid advancement of endoscopy, the detection rate for various types of gastric diseases has rapidly increased. Therefore, a rapid and effective Hp detection method is important. At present, various types of biopsy-based methods are used for Hp testing, and the gold standard for diagnosing Hp infection is histology or a combination of histology and culture.^5^ In recent years, most hospitals in China have used various histochemical and immunohistochemical staining methods for Hp detection, but the sensitivity and specificity of Hp tests vary. Different methods have their own advantages and disadvantages, but the reproducibility of the test results is often poor. Therefore, there is an urgent need for a staining method with high sensitivity, high specificity, and good reproducibility for the efficient detection of Hp infections in clinical practice. In this study, we combined our hospital’s last few years of Hp detection experience and effectively modified a traditional silver staining technique based on the characteristics of Hp to develop an efficient Hp test method (modified silver nitrate staining method). The staining results were compared with those of 3 commonly used Hp staining methods.

## MATERIALS AND METHODS

### Clinical Information

We selected gastric antrum and gastric angle mucosal biopsy tissues from 60 in patients that were archived in the Pathology Department of Zhongnan Hospital of Wuhan University from July to December 2020. Among these patients, there were 34 males and 26 females, and their ages ranged from 24 to 74 years, with a median age of 52 and 58 years in males and females, respectively. These 60 patients (defined as experiment group) underwent urea carbon-14-urea breath test (^14^C-UBT) tests and were confirmed to be positive for Hp infection. The tissues of the 60 patients were simultaneously sectioned. An Hp immunohistochemical assay, histochemical assay kit (methylene blue), and modified silver nitrate staining were used to measure the Hp infection positivity rate. At the same time, the gastric antrum and gastric angle mucosal biopsy tissues of 60 patients who tested negative for Hp infection by the urea ^14^C-UBT test were used as negative controls (defined as control group). Gastric ulcer and tumors were excluded from all patients enrolled in the study, including the experimental and control group.

### HE Staining

Experimental group and control group’s biopsy tissues were fixed in 10% neutral formalin followed by gradient dehydration, clearing, and paraffin embedding. Tissues were cut into 3-µm-thick serial sections, which in order to ensure the comparability of different methods in the same location, followed by routine dewaxing and hydration. This was followed by 5 minutes of hematoxylin staining and rinsing with running water. After that, hydrochloric acid-alcohol was used for 30 seconds of differentiation. Running water was used to wash the sections followed by blueing with lukewarm water for 30 seconds, and water-soluble eosin was then used for 30 seconds of staining. After dehydration and clearing, the sections were mounted in neutral resin.

### Hp Histochemical Assay Kit (Methylene Blue) Staining

An Hp staining assay kit (methylene blue) was used for histochemical staining (BaSO Diagnostics Inc. Zhuhai, BA4077). The manufacturer instructions were followed: serial sections were routinely dewaxed and hydrated. This was followed by staining with stomach Hp staining solution for 10 minutes. After washing, the sections were air-dried before mounting with neutral resin.

### Hp Immunohistochemistry

The 3-µm-thick serial sections were dried in a 70°C oven for 2 hours followed by dewaxing and hydration. After high-pressure antigen retrieval 30 minutes, the Dako immunohistochemistry device En-vision was used for 2-step detection. Sections were stained with an anti-Hp rabbit antibody (JY-0210, 1:200; JiaYuan, WuHan, China). After that, diaminobenzidine color development was carried out followed by applying hematoxylin for nuclear counterstaining. This was followed by dehydration and clearing before mounting of neutral resin.

### Hp Modified Silver Nitrate Staining Method

The 3-µm-thick serial sections were dewaxed and hydrated. After washing with distilled water, the sections were immersed in 1% silver nitration stain in the dark in a 60°C water bath for 60 minutes. Following that, distilled water was used to wash the sections before they were immersed in the developer solution, which was 2% gelatin (Fluca) and 0.12% hydroquinone in a 2:1 ratio. Following that, 60°C distilled water was used to rapidly rinse the sections on both sides. This was followed by an ethanol gradient for dehydration, clearing, and mounting with neutral resin.

### Statistical Analysis

The protein expression level was determined by densitometry of the bands using Image-pro Plus 6.0 analysis software. All statistical analyses were performed using SPSS15.0 software package. All values are expressed as mean±SD. Statistical analysis of the data uses analysis of variance, followed by *t* test. If *P*<0.05, the difference is considered significant.

### Ethics

This study was conducted in accordance with the requirements of the ethics committee at the Zhongnan Hospital of Wuhan University.

## RESULTS

### Comparison of Hp Morphology Detected Using the 3 Methods

**FIGURE 1:**
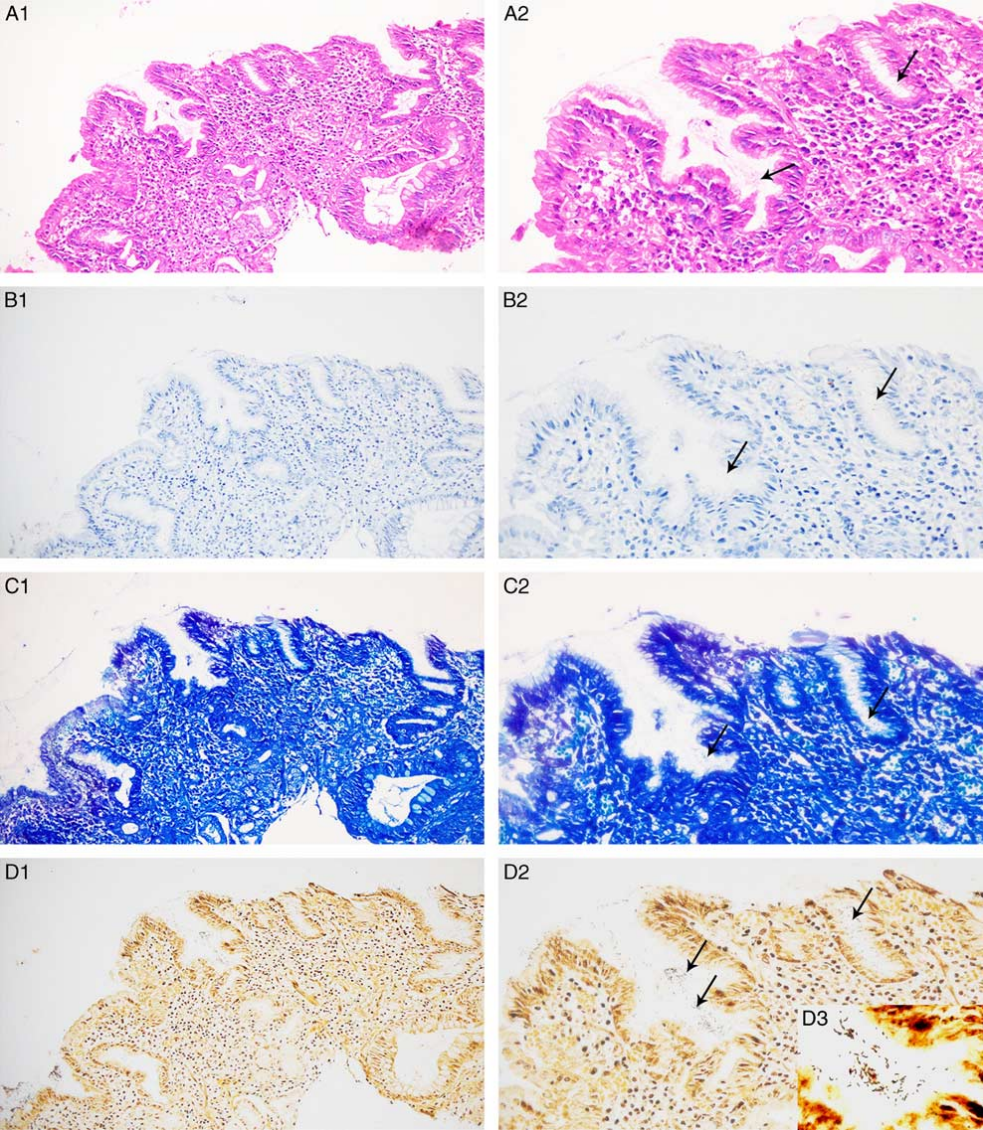
*Helicobacter pylori* morphology using the three detection methods. A1–A2, Hematoxylin and eosin stain. B1–B2, *Helicobacter pylori* immunostain. C1–C2, Methylene blue stain. D1–D3, Modified silver nitrate stain. Images for A1–D1 were obtained at ×40; for A2–D2 at ×400; and for D3 (inset) at ×1000.

Mucus distribution is common in gastric pits in biopsy tissues, and gastric pits in the gastric antrum and gastric angle are sites of Hp aggregation. Hematoxylin and Eosin (HE)-stained serial sections showed pink staining of mucus in the gastric pits. The presence of Hp could not be distinguished under ×200 magnification (Fig. 1A1), but blurry and slightly curved Hp rods were seen under ×400 magnification (Fig. 1A2, arrows).

The gastric pit background was clear under ×200 magnification after immunohistochemical staining, but Hp the immune response signal could not be distinguished (Fig. 1B1), while blurry S-shaped or long and brown positive Hp signals were seen under ×400 magnification, and clumpy or spherical brown positive Hp signals were seen locally (Fig. 1B2, arrows). Under the methylene blue stain, Hp in the gastric antrum and gastric angle biopsy tissues appeared blue, red blood cells appeared green, and the staining background appeared blue. The staining results under ×200 and ×400 were the same as those under immunohistochemical staining (Figs. 1C1, C2).

The gastric pit background was clean and clear under ×200 magnification when the modified silver nitrate staining method was used. As Hp appeared brownish-black after silver nitrate staining, the background contrast was significant and resolution was higher. Rod-shaped Hp and school-like aggregation could be observed under ×400 magnifications, and the Hp detection rate was significantly increased (Figs. 1D1, D2).

### Comparison of Hp Sensitivity and Specificity Between the 3 Methods

**FIGURE 2:**
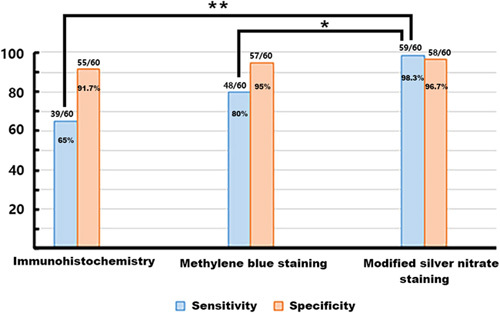
Comparison of *Helicobacter pylori* sensitivity and specificity between the 3 methods. The modified silver nitrate staining method has the highest sensitivity and specificity. *P<0.05, **P<0.01.

In the experimental group, the Hp sensitivities of immunohistochemistry, the methylene blue staining and modified silver nitrate staining were 65% (39/60), 80% (48/60), and 98.3% (59/60), respectively (Table 1). Analysis of the 3 methods showed that there were significant differences in sensitivity between the modified silver nitrate staining method and immunohistochemistry and methylene blue staining (*P*<0.05) (Fig. 2). On the 60 Hp negative controls (tested negative for Hp infection by the urea ^14^C-UBT test), the specificity of these 3 methods for detecting Hp infection were 91.7% (55/60), 95% (57/60), and 96.7% (58/60), respectively (Table 1). Different numbers of false positive cases were detected in the 3 methods. However, these false positive cases are different ones rather than the same samples. These phenomena showed that there is occasional appearance of false positive results in different methods. All the specificity of these 3 methods >90%, and there was no statistical difference between them (*P*>0.05) (Fig. 2).

**TABLE 1:** Comparison Among the Hp Detection Methods in the Sensitivity and Specificity

	Immunohistochemistry		Methylene Blue Staining		Modified Silver Nitrate Staining	
Standard	Hp (+)	Hp (−)	Hp (+)	Hp (−)	Hp (+)	Hp (−)
Experimental group (case)	39	21	48	12	59	1
Sensitivity	65% (39/60)	35% (21/60)	80% (48/60)	20% (12/60)	98.3% (59/60)	0.7% (1/60)
Control group (case)	5	55	3	57	2	58
Specificity	8.3% (5/60)	91.7% (55/60)	5% (3/60)	95% (57/60)	3.4% (2/60)	96.7% (58/60)

Experiment group were confirmed to be positive for Hp infection underwent urea ^14^C-UBT tests.

Control group were confirmed to be negative for Hp infection underwent urea ^14^C-UBT tests.

^14^C-UBT indicates carbon-14-urea breath test; Hp*, Helicobacter pylori*.

## DISCUSSION

Hp is a helical gram-negative rod that is present on the gastric mucosa.^6^ Previous studies have shown that Hp infection is an important cause of active gastritis.^7^ In addition, Hp infection is associated with gastric cancer in ∼50% of gastric cancer patients.^8^ Therefore, an effective method for detecting Hp in clinical practice is important for the prevention and control of gastrointestinal diseases that it causes. Clinical methods for the detection of Hp infection are classified as invasive and noninvasive.^5^,^9^ Different detection methods employ different principles, are suitable for different populations, and have their own advantages and disadvantages. The urea ^14^C-UBT non-invasive method is considered effective for diagnosing Hp infection without culture.^10^ The urea ^14^C-UBT method is simple, rapid, and noninvasive, and is suitable for preliminary screening of Hp infection in healthy subjects. Endoscopy is the main invasive method for obtaining biopsy tissues for further testing, while histologic tests are usually considered the gold standard for detecting Hp infection. With the rapid advancement of endoscopy techniques, biopsy tissue samples have become increasingly common. Hp infection has become a normal parameter in biopsy histopathology reports. Therefore, rapid and effective Hp detection methods are needed by laboratories.

Because of the presence of mucus in gastric mucosa biopsy tissues or HE staining itself, only blurry and concentrated pink clumps can be seen in HE-stained tissues at ×400 magnification, and it is difficult to clearly identify Hp morphology. Therefore, immunohistochemical or histochemical methods are required to confirm Hp infection for HE-stained sections.

Immunohistochemical detection methods are time consuming and relatively expensive. Their strengths include a clear background and high specificity, but they have weak positive signals and low sensitivity. When immunohistochemistry test results for severe Hp infection are relatively weak, patients with mild infections may have poor test results, causing the Hp missed diagnosis rate to be high. The commonly used histochemical staining kit (methylene blue) method is currently widely used in China. Its main characteristics are high sensitivity and specificity, fast staining, and simple operation, and it costs less than immunohistochemical methods. However, the background stain of methylene blue-stained mucosal tissues is blue, and Hp is stained as blue rods, which results in poor staining contrast. In addition, mucus tends to be stained blue, which increases the observation difficulty.

In this study, we combined various known silver nitrate staining methods and modified the concentration of the staining solution and ratio of staining solution to buffer to identify the best staining concentration and method. This staining method has been well-validated in our laboratory and has high sensitivity and specificity, a pale yellow mucosal tissue background, and the Hp appear as dark brownish-black rods, which has a large color difference with the background, facilitating identification. Therefore, this increases the detection rate of Hp and provides effective clues for clinical diagnosis. In addition, the cost of the modified silver nitrate method is lower than those of immunohistochemistry and histochemical staining kits, which greatly decreases the financial burden of patients. In the modified silver nitrate staining method, common laboratory reagent powders can be used to prepare fresh staining solutions using a simple method. This effectively solves the problem of reagent shelf-life, results in better control of the staining environment and staining process, and ensures staining quality. Modified silver nitrate staining requires around 70 minutes, which is much shorter than conventional immunohistochemical staining (Table 2).

**TABLE 2:** Morphologic Characteristics of Hp Detection in 3 Methods

Methods	Time (min)	Morphologic Characteristics	Advantages	Disadvantages
Immunohistochemistry	240	The nuclei of the biopsy tissue were blue; the Hp was brown-yellow, rod-shaped	The specificity is better, background cleaning, Hp morphology is obviously under the microscope	Longer dyeing time; The sensitivity is relatively poor
Methylene blue staining	30	The background is blue and the Hp is blue rod-shape too	The specificity and sensitivity are better, convenient and time saving	The blue Hp bacteria on the blue background. It is similar to the background, which is not easily to observe
Modified silver nitrate staining	70	The background is pale yellow and the Hp is dark brown rod-shape	The sensitivity and the specificity are the highest, saving time and easy to observe	To avoid light in the operations

Hp indicates *Helicobacter pylori*.

In addition, some researchers believe that in situ hybridization has better accuracy for Hp detection. In patients who lack gastric acid, Hp mostly appears as spherical because of antibacterial drugs and the growth of other bacteria in the stomach. Under this situation, it is difficult to accurately identify Hp through histochemical staining of biopsy tissue sections. In situ hybridization can be used to determine the morphology of infectious microorganisms and effectively detect Hp. However, in situ hybridization is expensive and time consuming, which limits its application for routine gastroscopy biopsy tissues. In this study, we compared different Hp detection methods. As the modified silver nitrate staining method is simple, has a short staining duration, low reagent costs, high reproducibility, and a clear and identifiable background, it can be widely used as a detection method in clinical practice.

## MAIN POINTS

1. This study improved the traditional methods for detecting HP and obtained a practical clinical detection method.
2. Hp modified silver nitrate staining method has both high sensitivity and specificity compare to the traditional methods.
3. As the modified silver nitrate staining method is simple, has a short staining duration, low reagent costs, high reproducibility, and a clear and identifiable background, it can be widely used as a detection method in clinical practice.
